# Preclinical evidence of multiple mechanisms underlying trastuzumab resistance in gastric cancer

**DOI:** 10.18632/oncotarget.7575

**Published:** 2016-02-22

**Authors:** Chiara Arienti, Michele Zanoni, Sara Pignatta, Alberto Del Rio, Silvia Carloni, Michela Tebaldi, Gianluca Tedaldi, Anna Tesei

**Affiliations:** ^1^ Biosciences Laboratory, Istituto Scientifico Romagnolo per lo Studio e la Cura dei Tumori (IRST) IRCCS, Meldola, Italy; ^2^ Institute of Organic Synthesis and Photoreactivity (ISOF), National Research Council (CNR), Bologna, Italy; ^3^ Innovamol Srls, Modena, Italy

**Keywords:** trastuzumab resistance, gastric cancer, HER family receptors, HER signaling pathways, IQGAP1

## Abstract

HER2-positive advanced gastric cancer patients frequently develop resistance to trastuzumab through mechanisms still poorly understood. In breast cancer, other members of the HER-family are known to be involved in trastuzumab-resistance, as is overexpression of the scaffold protein IQGAP1. In the present work, we investigated acquired resistance to trastuzumab in gastric cancer experimental models. Trastuzumab-resistant (HR) subclones derived from 3 HER2-overexpressing gastric cancer cells were generated and characterized for alterations in HER2-signaling mechanisms by next-generation sequencing, immunohistochemical, western blot and qRT-PCR techniques, and molecular modeling analysis. All subclones showed a reduced growth rate with respect to parental cell lines but each had a different resistance mechanism. In NCI N87 HR cells, characterized by a marked increase in HER2-signaling pathways with respect to the parental cell line, trastuzumab sensitivity was restored when IQGAP1 expression was silenced. AKG HR subclone showed higher HER3 protein expression than the parental line. High nuclear HER4 levels were observed in KKP HR cells. In conclusion, our study revealed that high IQGAP1 expression leads to resistance to trastuzumab in gastric cancer. Furthermore, 2 new mutations of the HER2 gene that may be involved in acquired resistance were identified in AKG HR and KKP HR subclones.

## INTRODUCTION

Gastric cancer is the fourth most common malignant disease and the second leading cause of cancer-related death worldwide [1]. In Europe, it is the fifth most common cancer among men and women, representing about 23% of all cancers. Depending on tumor characteristics and stage [2], treatment modalities include a combination of surgery, chemotherapy generally based upon a platinum-fluoropyrimidine doublet, and radiation therapy [3, 4]. Although fluorouracil (5-FU)-based regimens have proven feasible and effective in the treatment of solid tumors, their therapeutic effect is unsatisfactory in advanced gastric cancer, *i.e*., 7-51% overall response rate and 6 to 12-month median survival [5–7]. Thus, various combination regimens have been developed.

Trastuzumab, a monoclonal antibody targeting human epidermal growth factor receptor 2 (HER2), induces antibody-dependent cellular cytotoxicity and inhibits HER2-mediated signaling by binding the extracellular domain of HER2. Amplification of the *HER2* gene is observed in 20%-30% of gastric and gastroesophageal junction cancer [8–12] and is indicative of a poor prognosis, as recently highlighted in the systematic meta-analysis by Jorgensen et al. [13]. In 2010, the phase III ToGA trial showed the superiority of trastuzumab plus chemotherapy (based on a cisplatin-fluoropyrimidine doublet) in patients with HER2-positive metastatic gastric cancer over chemotherapy alone in terms of response rate, progression-free survival (PFS) and overall survival (OS) [14]. These results led to the approval of trastuzumab as the first molecular targeted therapy for gastric cancer. However, subsequent clinical trials (TYTAN8 and LOGiC9) failed to show a survival advantage with the use of another anti-HER2 treatment, lapatinib [15]. Overall, the efficacy of HER2-targeted agents has proven more limited and unsatisfactory than originally expected because the majority of patients with gastric cancer develop acquire resistance to treatment [16]. In particular, it has been observed that, whilst few patients with HER2-positive advanced gastric cancer exhibit primary resistance to trastuzumab, all acquire resistance after a relatively short period of time (median PFS 6.7 months) [17], as already observed in HER2-positive breast cancer patients. The identification of mechanisms underlying treatment resistance would thus enhance the benefit from HER2-targeted therapy in patients with HER2-positive gastric cancer.

The etiology of resistance to HER2-directed therapies has been widely investigated in breast cancer [18–22]. Several molecular mechanisms underlying acquired resistance to HER-2 inhibitors have been described, including the activation of c-Src tyrosine kinase [20], HER3 upregulation [23], activating mutations in the p110a subunit of PI3K (PIK3CA) [24], and enhanced HER-ligand autocrine signaling [25]. It has also been proven that resistance to HER2-targeted therapy can trigger genetic alterations of receptor tyrosine kinases (RTKs), leading to the activation of downstream signaling targets and alternative pathways to compensate for HER-2 inhibition [26, 27]. Numerous studies have concluded that induction of the HER3 pathway is one of the reasons underlying this type of resistance [28–30]. Moreover, Mohd Nafi et al. observed that HER4 activation, cleavage and nuclear translocation influence sensitivity and resistance to trastuzumab in HER2-positive breast cancer [31].

A recent study reported that IQGAP1, a scaffold protein of 189-kDa ubiquitously expressed in all human tissues, governs HER2 expression, phosphorylation and signaling in breast cancer cell lines [32]. Moreover, IQGAP1 protein is overexpressed in squamous cell [33] and hepatocellular [34] carcinoma, astrocytoma [35], and aggressive forms of gastric cancer [36]. In particular, White et al. [37] showed that IQGAP1overexpression is correlated with trastuzumab-induced resistance in breast cancer cell lines. However, its involvement in resistance to trastuzumab in gastric cancer has never been investigated. In the present work we investigated mechanisms of resistance induced by trastuzumab in *in vitro* experimental gastric cancer cell lines rendered resistant to the antiproliferative effect of the drug.

## RESULTS

### Baseline expression and mutational status of HER2, -3 and -4 receptors in a panel of established human gastric cancer cell lines

**Figure 1:**
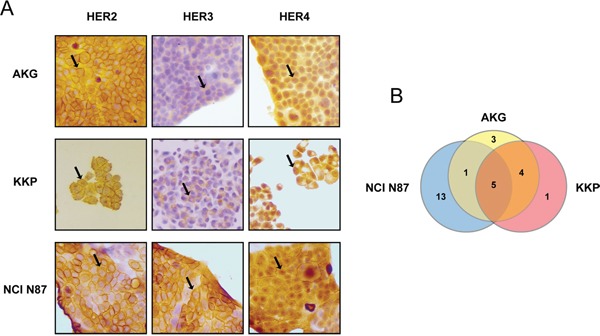
Baseline expression and mutational status of HER2, -3 and -4 receptors in a panel of established human gastric cancer cell lines **A.** HER2, HER3 and HER4 staining by IHC in gastric cancer cell lines AKG, KKP and NCI N87. Sample reactivity was evaluated by light microscopy (×200 magnification) by two independent observers. Marker positivity was evaluated in a semiquantitative manner, as described in the *Materials and Methods* section. **B.** Venn-diagram comparison of gene variants across the three cell lines. The numbers refer to the number of somatic mutations affecting HER2, -3 and -4.

Positivity to HER2, -3 and -4 proteins and their cellular localization in the human gastric cell lines NCI N87, AKG and KKP was assessed by immunohistochemistry (Figure 1A). HER2, -3 and -4 receptors were highly expressed in all 3 cell lines, albeit with a different diffusion pattern. In particular, HER2 was highly expressed in NCI N87 with a diffuse plasma membrane and cytosolic staining pattern. HER3 was also highly expressed in NCI N87 cells (∼95% of positive cells) in both the plasma membrane and cytosol. In addition, HER3 was expressed in KKP and AKG cells, albeit to a lesser degree (∼40% and ∼30% of positive cells, respectively), whereas its staining pattern was mainly restricted to the cytosol. Finally, HER4 protein was mainly localized in the nuclei and cytoplasm of AKG cells.

We used next generation sequencing (NGS) to search for genomic alterations that might predispose to a different response to treatment with trastuzumab (average depth of 2779.73 and 99.7% of targets with a minimum coverage of 50). We first investigated whether the different cell lines harbored genetic alterations *of HER2, HER3 or HER4* genes and which, if any, were common to AKG, KKP and NCI N87 (Figure 1B). All 3 cell lines showed several alterations in the gene sequences investigated, only 12 of which are not annotated in the dbSNP and COSMIC databases. In particular, 4 of these were exonic variants (Tables 1 and 2). Notably, only 5 gene variants were common to all 3 lines, *i.e*. HER3 variants c.-211delCT and c. C2270A; and HER4 variants IVS17-60delAG, IVS17-102insG and IVS7-7delT (Figure 1B and Table 1).

**Table 1:** Genetic variants shared in parental gastric cancer cell lines

Cell lines	gene	variant	localization	variant effect	protein change	annotation	Functional prediction score (HVAR, SIFT, MutationAssessor)
**AKG KKP**	HER2	c.C3508G	exonic	nonsynonymous SNV	p.P1170A	rs1058808	P, D, M
	HER3	c.-277T>C	upstream	-	-	rs7297175	-
		IVS2+8A>T	intronic	-	-	rs2271194	-
	HER4	IVS16-23insA	intronic	-	-	rs202070359	-
**AKG NCIN87**	HER4	IVS12-15T>C	intronic	-	-	rs4673628	-
**AKG KKP NCIN87**	HER3	c.-211delCT	UTR5	-	-	-	-
		c.C2270A	exonic; splicing	nonsynonymous SNV	p.T757K	-	D, D, N
	HER4	IVS17-60delAG	intronic	-	-	rs146953835	-
		IVS17-102insG	intronic	-	-	rs76332141	-
		IVS7-7delT	intronic	-	-	rs67894136	-

HVAR outputs: P, potentially deleterious; D, probably deleterious; SIFT outputs: D, deleterious; MutationAssessor outputs:, N, neutral; M, medium; -, unknown

**Table 2:** Genetic variants not shared by parental gastric cancer cell lines

Cell lines	gene	variant	localization	variant effect	protein change	annotation	Funtional prediction score (HVAR, SIFT, MutationAssessor)
**AKG**	HER2	IVS8-7T>C	intronic (7bp)	-	-	-	-
		c.T3182C	exonic	nonsynonymous SNV	p.L1061P	rs141142822	P, T, N
	HER3	c.-195delCA	UTR5	-	-	-	-
**KKP**	HER2	c.T2709G	exonic	nonsynonymous SNV	p.S903R	-	D, D, H
**NCIN87**	HER2	c.A2698C	exonic	nonsynonymous SNV	p.T900P	-	D, D, M
		c.C2692G	exonic	nonsynonymous SNV	p.R898G	-	D, D, L
		c.C2704A	exonic	nonsynonymous SNV	p.Q902K	-	D, D, N
		c.C2689G	exonic	nonsynonymous SNV	p.R897G	-	D, T, N
		c.A2705G	exonic	nonsynonymous SNV	p.Q902R	-	D, D, N
	HER3	c.A3355T	exonic	nonsynonymous SNV	p.S1119C	-	D, T, N
		c.G2606A	exonic	nonsynonymous SNV	p.S869N	rs143021252	B, T, N
		IVS27-7C>T	intronic	-	-	rs812826	-
	HER4	IVS24-7delCTTT	splicing	-	-	rs138150601	-
		IVS13-12A>T	intronic	-	-	rs78812564	-
		IVS25-53delC	intronic	-	-	rs142227938	-
		IVS21+81insA	intronic	-	-	rs141267844	-
		IVS16-18delT	intronic	-	-	-	-

HVAR outputs: B, benign; P, potentially deleterious; D, probably deleterious; SIFT outputs: T, tolerated; D, deleterious; MutationAssessor outputs: N, neutral; L, low; M, medium; H, high; -, unknown

A pairwise comparison of the 3 cell lines revealed that AKG and KKP cell lines shared the highest number of gene alterations, one in *HER2*, 2 in *HER3* and one in *HER4* (Table 1). Conversely, NCI N87 showed the highest number of genetic variants (13) relating to all 3 HER receptors (5 variants in *HER2*, 3 in *HER3* and 5 in *HER4*) that were not found in AKG or KKP (Table 2).

### Generation of trastuzumab-resistant subclones

**Figure 2:**
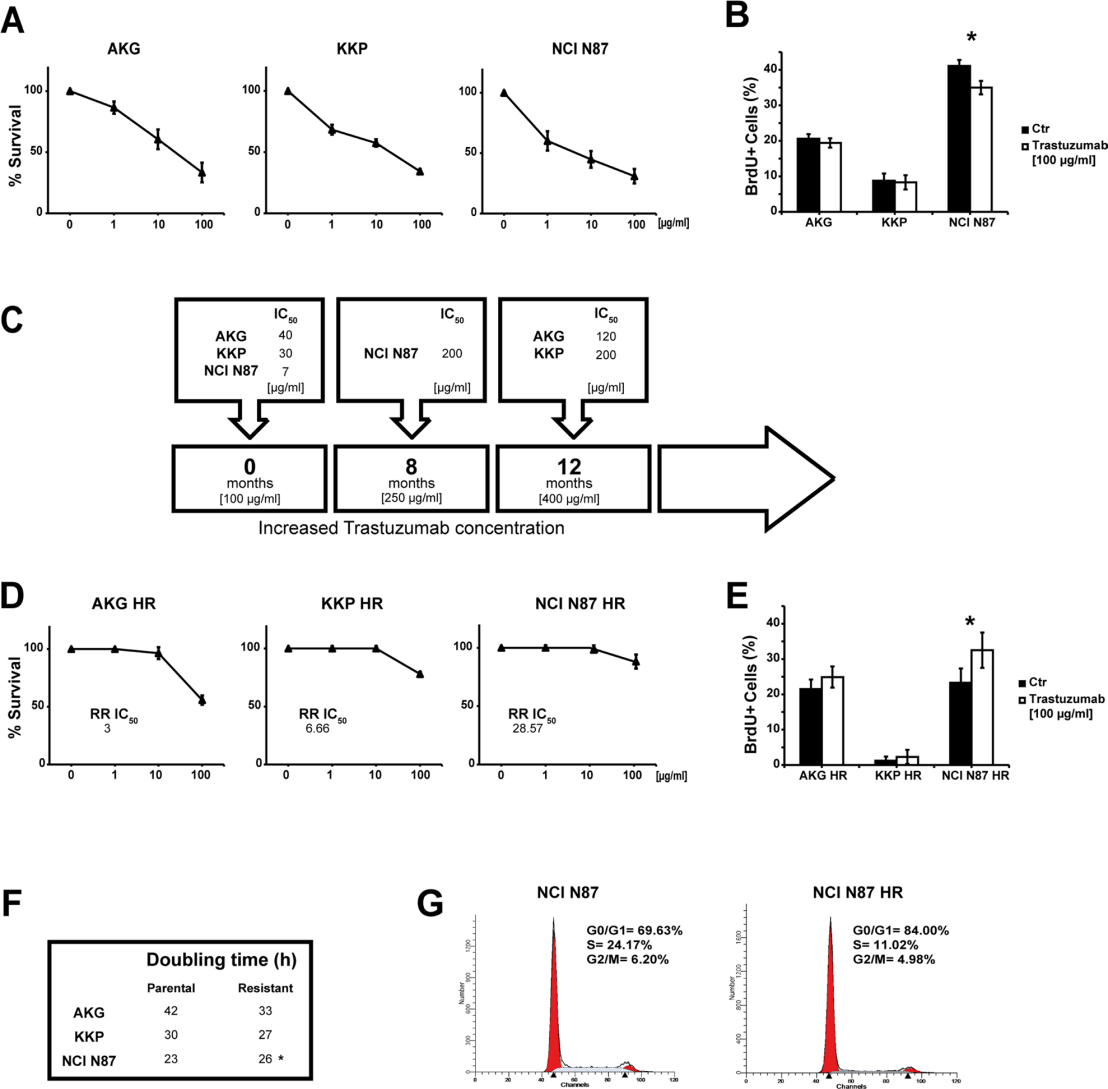
Induction of trastuzumab-resistance in gastric cancer cell lines **A.** Trastuzumab sensitivity curves in parental cell lines evaluated by clonogenic assay. Each point indicates the mean of at least three experiments. The standard deviation never exceeded 5%. **B.** Percentage (median value) of BrdU-positive cells after trastuzumab treatment (100 μg/ml). Values are the mean ± SD of three independent experiments. * significance at p<0.05 by t-test. **C.** The induction timeline of trastuzumab resistance. Resistant cells were generated by continuous treatment with trastuzumab for more than 8 months. **D.** Trastuzumab sensitivity curves in resistant cell lines evaluated by clonogenic assay. Each point indicates the mean of at least three experiments. Standard deviation never exceeded 5%. **E.** Percentage (median value) of BrdU-positive cells after trastuzumab treatment (100 μg/ml). Values are the mean ± SD of three independent experiments. * significance at p<0.05 by t-test. **F.** Doubling times of parental and resistant cell lines. **G.** Cell cycle analysis of NCI N87 and NCI N87 HR by flow cytometry. Data are expressed as a percentage of distribution in each cell-cycle phase.

All of the cell lines were sensitive to trastuzumab, as confirmed by the clonogenic assay in which IC_50_ values were lower than the peak plasma concentration of the drug (Figure 2A). In particular, NCI N87, the cell line harboring the highest number of HER2 variants, was the most sensitive to the cytotoxic action of trastuzumab (IC_50_ value of 7 μg/ml), whereas the AKG cells (3 gene variants) were the most resistant (IC_50_ = 40 μg/ml). The incorporation of BrdU after a 72-h treatment with the drug confirmed these data. In fact, after treatment with 100 μg/ml of trastuzumab, NCI N87 showed a lower incorporation of BrdU than that of untreated cells (35% and 42%, respectively), while no substantial change in cell proliferation was seen in AKG (Figure 2B).

We generated trastuzumab-resistant (HR) subclones derived from the above gastric cancer cell lines to investigate the mechanisms underlying acquired resistance to trastuzumab. Starting from the peak plasma concentration of 100 μg/ml, all cell lines were exposed to gradually increasing concentrations of trastuzumab for a period of 8-12 months. We thus obtained trastuzumab-resistant subclones that were capable of growing in culture medium containing a drug concentration of 250 μg/ml for the NCI N87 HR subclone and 400 μg/ml for the AKG HR and KKP HR subclones (Figure 2C).

The resistant phenotype was stable and all subclones showed IC_50_ values higher than the peak plasma concentration of the drug ranging from 120 μg/ml (AKG HR) to 200 μg/ml (KKP HR and NCI N87 HR) (Figure 2C). We also evaluated the relative resistance IC_50_ index (RR IC*_50_*) of each subclone obtained (Figure 2D). Notably, the data revealed that the subclone with the highest RR IC_50_ value, NCI N87 HR, was obtained from the cell line with the highest number of genetic variants. In addition, trastuzumab was found to stimulate proliferation in all subclones, significantly so for NCI N87 HR cells (p<0.05) (Figure 2E).

We also observed changes in doubling times that were cell line-dependent (Figure 2F), *e.g.* AKG HR cells grew more rapidly, albeit not significantly, than those of the parental line. KKP and its subclone KKP HR showed similar growth, while NCI N87 HR grew significantly slower than its parental line (p<0.05).

The resistant subclone of NCI N87 displayed a different cell distribution in cell cycle phases compared to parental cells. In particular, an increase of cells in G0/G1 phase was observed in NCI-N87 HR (84.0%) compared to NCI N87 cells (69.63%), whereas a lower percentage of S-phase cells was found in the HR subclone than in the parental line (11.02% NCI N87 HR *vs*. 24.17% NCI N87) (Figure 2G).

### Different HER2 signaling modulation in HR subclones

**Figure 3:**
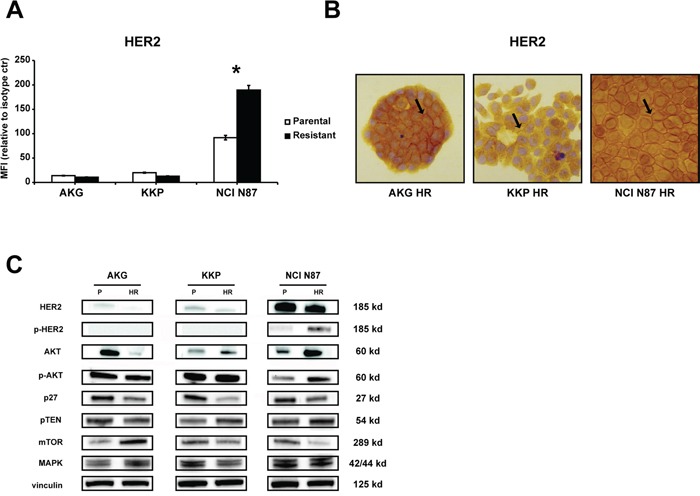
Characterization of trastuzumab targeting in resistant subclones: AKG HR, KKP HR and NCI N87 HR cells **A.** HER2 protein levels on the cell surface were quantified by flow cytometry and expressed as mean fluorescence intensity (MFI) relative to isotype control. Statistical significance was denoted as * p<0.05. **B.** HER2 staining by IHC in gastric cancer cell lines. Sample reactivity was evaluated by light microscopy (× 200 magnification) by two independent observers. Marker positivity was evaluated in a semiquantitative manner, as described in the *Materials and Methods* section. **C.** Western blotting showed HER2, p-HER2, AKT, p-AKT, p27, PTEN, mTOR and MAPK protein expression in parental (P) and resistant (HR) cell lines. Vinculin expression indicated equal loading. All gels were run under the same experimental conditions and the experiments were repeated 3 times. The representative images were cropped and shown.

We quantified HER2 expression levels by flow cytometry and western blot analysis to verify whether its expression was modified in HR subclones (Figure 3). Flow cytometric analysis revealed an increase in HER2 membrane levels in NCI N87 HR cells with respect to parental cells (Figure 3A). Furthermore, immunohistochemistry analysis showed that HER2 was highly expressed in NCI N87 HR cells (∼95% positive cells) which had both membrane and a cytoplasmic positivity (Figure 3B). We also detected a marked increase in p-HER2, AKT, p-AKT and MAPK protein levels in NCI N87 HR with respect to NCI N87 (Figure 3C). Protein levels of p-HER2 and of molecules involved in HER2 signaling were also analyzed by Western blot in the other cell lines. An increase in AKT expression was detected in KKP HR cells, while MAPK and p27 expression levels were significantly lower than those of the parental cell line. In AKG HR cell line, we observed an increase in mTOR and MAPK protein expression and a sharp decrease in AKT protein expression with respect to parental AKG cells.

### Knockdown of IQGAP1 inhibits HER2-stimulated NCI-N87 HR cell growth

**Figure 4:**
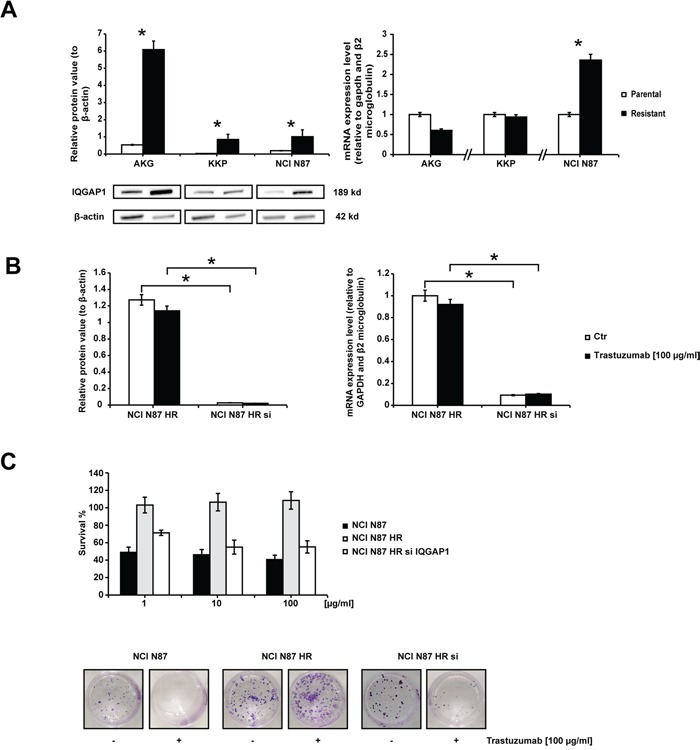
Trastuzumab sensitivity in subclone NCI N87 HR restored by IQGAP1 knockdown **A.** IQGAP1 expression. The left panel shows the western blot of IQGAP1 in gastric cancer cell lines. β-actin expression indicated equal loading. Densitometric quantification of total IQGAP1 was calculated using Quantity One Software. All gels were run under the same experimental conditions and the experiments were repeated 3 times. The representative images were cropped and shown. The right graph shows IQGAP1 mRNA expression levels in resistant cells quantified with respect to parental cells and normalized to GAPDH and β2 microglobulin. Data are presented as mean +- SD. * p<0.05. **B.** IQGAP1 silencing. IQGAP1 protein expression levels (left graph) and IQGAP1 mRNA levels (right graph) in NCI N87 HR and NCI N87 HR IQGAP1-silenced before and after a 72 h-exposure to 100 μg/ml of trastuzumab. Western blot analysis of IQGAP1 was normalized to β-actin. IQGAP1 mRNA levels were measured by Real Time PCR and normalized to GAPDH and β2 microglobulin. Values are the mean ± SD of three independent experiments. **C.** The effect of IQGAP1 knockdown in NCI N87 HR cells was investigated in colony formation experiments carried out 72 h after transfection. The upper panel illustrates relative growth curves (means +- SD) and the bottom panel shows representative colony photos.

IQGAP1 gene and protein expression were analyzed in all cell lines to verify their involvement in trastuzumab-related resistance (Figure 4). A different modulation of the protein was observed in the 3 parental cell lines, AKG cells showing the highest IQGAP1 expression and KKP the lowest (Figure 4A). This expression pattern was confirmed by gene expression analysis (data not shown). We also observed an increase in IQGAP1 protein expression of HR-resistant subclones with respect to parental cells (p<0.05). However, trastuzumab-resistant NCI N87 cells were the only subclones to show an increase in IQGAP1 gene expression levels with respect to parental cells (expression value 2.5-fold higher than that of NCI N87).

The influence of IQGAP1 on trastuzumab resistance was evaluated by transfecting siRNAs against IQGAP1 into NCI N87 HR cells, the subclone with the highest drug resistance phenotype (also confirmed by its RR value). Gene silencing induced a total block in protein synthesis and a dramatic decrease (up to 90%) in mRNA expression (Figure 4B). IQGAP1-silenced NCI N87 HR cells exposed to different concentrations of trastuzumab for 144 h regained a certain degree of sensitivity to trastuzumab, reaching an IC_50_ value of 110 μg/ml. Furthermore, the RR decreased from 28.57 to 15.71, indicating an increase in sensitivity to trastuzumab (Figure 4C). These data were further supported by the results from a colony formation assay showing a reduction of about 55% in the number of colonies when IQGAP1-silenced NCI N87 HR cells were exposed to trastuzumab 100 μg/ml for 14 days.

### Analysis of HER3 and HER4 protein expression in HR subclones

**Figure 5:**
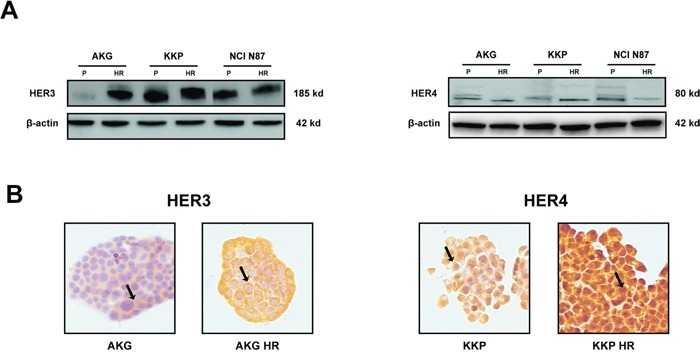
Characterization of resistant subclones: AKG HR, KKP HR and NCI N87 HR cells **A.** HER3 and HER4 protein levels in parental cell lines (P) and their derivative subclones (HR) evaluated by western blot. The representative images were cropped and shown. Statistical significance was denoted as * p<0.05. **B.** HER3 and HER4 staining by IHC in AKG and KKP cells, respectively and in their derivative resistant subclones. Sample reactivity was evaluated by light microscopy (× 200 magnification) by two independent observers. Marker positivity was evaluated in a semiquantitative manner, as described in the *Materials and Methods* section.

HER3 and HER4 expression levels in the plasma membrane were quantified by western blot analysis and immunohistochemistry to evaluate their role in the acquired resistance to trastuzumab (Figure 5). Protein expression detected by western blot revealed increased HER3 and decreased HER4 protein levels in AKG HR cells compared to parental cells. KKP HR subclone showed higher levels of HER4 than KKP cells. Finally, NCI N87 HR only showed significantly lower HER4 expression than parental cells (Figure 5A). HER3 was also highly expressed in about 95% of AKG HR cells, with cytoplasmic positivity. Furthermore, immunohistochemistry analysis showed that HER4 was localized exclusively in the nuclei and cytoplasm of all trastuzumab-resistant subclones and, in particular, was highly expressed in KKP HR (Figure 5B).

### Relationship between specific genetic variations and change in HER2 and HER3 receptor structure

**Figure 6:**
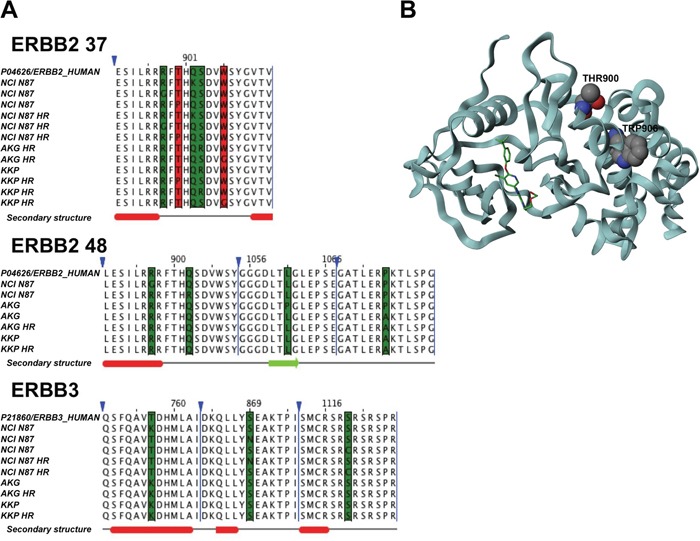
Comparison of sequence alignment between parental and trastuzumab resistant subclones **A.** Sequence alignments of HER2 37, HER2 48 isoforms and HER3 highlights mutations in the cell lines under investigation. The red columns represent mutations associated with resistant cells, while green columns represent other kinds of mutations. Triangles denote hidden columns in the sequence. Secondary structure prediction denotes α-helix (red) and β-strand (green). **B.** The crystal structure of the kinase domain of HER2 (HER2-KD) in complex with SYR127063 (PDB code 3PP0). The ligand (green) binds to the HER2 ATP binding site. The mutations that confer resistance are located in residues 900 and 906 (in CPK notation) in a region located at the C-terminal lobe of the HER2-KD, which is predominantly α-helical.

We analyzed the exonic variants of target genes detected by NGS to investigate their role in the onset of resistance to trastuzumab (Figure 6). None of the cell lines showed exonic genetic variants for HER4 or IQGAP1 genes. In addition, no intronic variants were found in the IQGAP1 gene (data not shown). Once again, the cells showing the highest number of variants of all parental cells or subclones were NCI N87, which was also the most sensitive to trastuzumab. In particular, 5 mutations were located in a region in the predominantly α-helical C-terminal lobe between residues 898 and 906 of HER2 isoform 37 and between residues 897 and 902 of HER2 isoform 48. Its resistant subclone, NCI N87 HR, did not acquire new genetic variants. Conversely, this subclone showed the loss of 3 variants with respect to the parental line. In particular, the genetic variants were located in clusters between residues 897 and 902 in the C-terminal lobe of HER2 isoform 48, and the mutation in position 759 belonging to the N-terminal lobe of HER3. Conversely, the trastuzumab-resistant subclones KKP HR and AKG HR acquired one and 2 genetic variants, respectively, compared to their parental cell lines, all located between residues 898 and 906 in a region in the predominantly α-helical C-terminal lobe of HER2 isoform 37 (Figure 6A). The crystal structure of the kinase domain of HER2 (HER2-KD) in complex with SYR127063 (PDB code 3PP0) is shown in Figure 6B.

## DISCUSSION

In gastric cancer patients, HER2/neu gene expression is an independent prognostic factor, and overexpression of the HER2 protein is correlated with poor prognosis and short-term survival [38, 39]. The effectiveness of trastuzumab and its subsequent approval as first-line treatment for HER2-overexpressing metastatic gastric cancer confirmed the importance of this receptor in gastric cancer. However, as already observed in other tumors, the majority of patients who initially show sensitivity to trastuzumab develop resistance within one year [17]. Aberrant HER2 activity and the activation of the HER2 receptor in human gastric tumors leads to receptor heterodimerization, mainly with HER3 and HER4 receptors [40], triggering a complex signal transduction cascade that modulates cancer cell survival, proliferation, mobility and invasiveness [41].

The main aim of our work was to investigate resistance mechanisms to trastuzumab in preclinical models of human gastric cancer. For this purpose we created trastuzumab resistant subclones starting from 3 HER2-overexpressing gastric cancer cell lines (AKG, KKP and NCI-N87 cells) with a high sensitivity to trastuzumab. These lines also expressed other HER family receptor members and showed genetic variants of HER2, HER3 and HER4.

After 8-12 months' exposure to increasing concentrations of trastuzumab, we successfully obtained trastuzumab-resistant AKG HR, KKP HR and NCI N87 HR subclones that grew in culture medium supplemented with high concentrations of the drug (up to 400 μg/ml). Cell growth curves, BrdU incorporation and cell cycle analyses revealed that the biological features of trastuzumab-resistant cells differed from those of parental cells. In particular, the NCI N87 HR subclone grew more slowly, had a lower proliferative activity and showed a higher percentage of cells in G_0_/G_1_ phase and a lower percentage in S phase than NCI N87 cells. Similar results were reported by Zou et al. [42] who were the first group to obtain a trastuzumab-resistant subclone of NCI-N87 (N87 NCI/TR). In addition, our flow cytometry analyses revealed an increase in membrane HER2 levels only in the NCI N87 HR subclone with respect to its parental cells. We also detected a marked increase in p-HER2, AKT, p-AKT and MAPK protein levels and a reduction in p27 protein expression in NCI N87 HR cells. These results are in agreement with those from previous studies in which trastuzumab was reported to inhibit HER2+ tumor growth by stimulating endocytosis and degradation of the receptor, with subsequent impairment of downstream signaling through PI3K/AKT and MAPK cascades [43]. It was also recently reported that increased PI3K/AKT and MAPK cascade signaling inhibits p27 expression [43–46].

White et al. revealed that IQGAP1 governs trastuzumab function in HER2-overexpressing breast cancer. In particular, they reported that, in IQGAP1-silenced breast cancer cells, trastuzumab increased its capacity to decrease HER2 expression and HER2-stimulated activation of the PI3K/AKT cascade [37].

In the present work we showed that IQGAP1 knockdown in gastric cancer leads to the abrogation of trastuzumab resistance and to restored drug sensitivity. In particular, IQGAP1 protein levels in all trastuzumab resistant subclones were higher than those of parental cells, although no genetic variants were detected in the different cell lines used. However, the restoral of trastuzumab sensitivity through IQGAP1 silencing was only observed in NCI N87 HR cells which showed a strong activation of PI3K/AKT and MAPK signaling cascades, both features of IQGAP1-mediated trastuzumab resistance [37].

Multiple factors influence the resistance to molecular-targeting drugs and several studies have hypothesized that acquired resistance to trastuzumab might also be due to the alteration of the signaling cascade induced by HER3 and HER4 receptors [20, 23, 24]. Our models showed high levels of both receptors. In particular, AKG HR cell line expressed higher protein levels of HER3 than the parental line, mainly in the cell membrane. This finding is in agreement with data reported by Ma et al. [47] who considered HER3 overexpression to be a mechanism of resistance to trastuzumab. Increased HER3 expression also appears to promote both PI-3 K7Akt signaling and Scr kinase activity [48]. In our study, KKP HR cells expressed high levels of nuclear HER4, confirming recent findings about the involvement of HER4, especially in terms of its activation, cleavage and nuclear translocation, in resistance to trastuzumab in breast cancer cell lines [31, 49].

We also investigated the presence of genetic variants potentially involved in acquired trastuzumab resistance. No genetic variants of the IQGAP1 gene were found in any of the studied cell lines. Furthermore, NGS analysis revealed that the resistant clone NCI N87 HR did not acquire additional gene variants with respect to the parental line. Conversely, KKP HR and AKG HR acquired one and two genetic variants, respectively, compared to their parental cell lines, and all were located in the C-terminal lobe of HER2, in a portion of the molecule called αF-helix. αF-helix, a highly hydrophobic component located in the middle of the C-lobe, plays a central role in anchoring key hydrophobic motifs. In particular, it forms the base of C- and R-spines, two motifs previously described by Kornev et al., [50, 51] which coordinate the N- and C-lobe movements of the kinase domain in the active conformation of the protein [52]. Given that both motifs are highly conserved through different types of active protein kinases, the assembly and anchorage of the spines to αF helix could be an important regulatory element. Furthermore, the activation loop, another important portion of the kinase domain, is firmly anchored to the hydrophobic αF helix. This is the most flexible part of the activation segment and requires phosphorylation to activate and increase the enzymatic activity of protein kinases, including ErbB family members [53, 54]. The genetic variants detected by our NGS analysis have never been reported before and may serve to maintain the active conformation of the HER2 receptor.

In conclusion, our study provides evidence of the existence of different mechanisms of resistance to trastuzumab in human gastric cancer. We also discovered that IQGAP1 is involved in trastuzumab resistance in gastric cancer cell lines and identified 2 new mutations of the HER2 gene that may be correlated with acquired resistance to the drug. Further studies are needed to explore these issues.

## MATERIALS AND METHODS

### Cell lines

The study was performed on two cell lines (AKG, KKP) derived from human gastric adenocarcinoma (intestinal type), established and characterized in our laboratory [55, 56], and one commercial cell line obtained from a liver metastasis of a well differentiated gastric carcinoma (NCI-N87) and purchased from the American Type Culture Collection (ATCC, Rockville, MD, USA). Cell lines were maintained as a monolayer at 37°C and subcultured weekly. The culture medium was composed of DMEM/Ham's F12 (1:1) supplemented with fetal calf serum (10%), glutamine (2 mM), non-essential amino acids (1%) (Mascia Brunelli S.p.A., Milan, Italy), and insulin (10 mg/ml) (Sigma-Aldrich, St. Louis, MO, USA). Cells were used in the exponential growth phase in all experiments.

### Doubling time

For growth analysis, cells were plated in 12-well plates in triplicate at a concentration of 2 × 10^4^ cells/well. Cells were collected and counted for the first 7 days after plating. Trastuzumab (*Herceptin*^®^) was purchased by the Oncology Pharmacy of our institute (IRST IRCCS). Proliferation doubling time was determined by the following formula: log2 (Cv/Cs), where Cv is the number of viable cells at harvest and Cs is the number of cells seeded. The sum of all previous population doublings determined the cumulative population doubling level at each passage. The Trypan blue exclusion test was used to evaluate the percentage of viable cells, which always exceeded 98% for the duration of the experiments.

### Generation of trastuzumab-resistant subclones

We induce trastuzumab resistance by culturing trastuzumab-sensitive gastric cancer cell lines in the presence of progressively increasing doses of trastuzumab over a period of 12 months. The final concentration of trastuzumab used was 250 μg/ml for the trastuzumab-resistant subclone NCI-N87 HR and 400 μg/ml for the subclones AKG HR and KKP HR.

### Immunohistochemistry

Cells were seeded in sterile culture slides (BD, Falcon, New Jersey, USA) and cultured in a humidified CO2 incubator for 72 h. They were then fixed in 4% (v/v) paraformaldehyde for 20 min and blocked for endogenous peroxidase activity with a 3% hydrogen peroxide solution. Antigen unmasking was performed using citrate buffer pH 6 for 40 min at 98.5°C. Rabbit monoclonal anti-human antibodies for HER3 (Cell Signaling Technology, Inc., Danvers, MA, USA) and HER4 (Santa Cruz Biotechnology, Dallas, Texas, USA) were used at a dilution of 1:250. Mouse monoclonal anti-human antibody for HER2 (Dako Corporation, Carpenteria, CA, USA) was used at a dilution of 1:100. Antibodies were incubated for 60 min at room temperature. Slides were washed with phosphate buffered saline (PBS), incubated with a universal biotinylated secondary antibody for 15 min and rinsed in PBS. They were then incubated with streptavidin-peroxidase conjugate (LSAB + Kit; Dako Corporation) for 15 min. Slides were rinsed again in PBS and antibody binding was detected by staining with diaminobenzidine/hydrogen peroxidase chromogen solution (DAB + liquid substrate–chromogen solution; Dako Corporation). Finally, the sections were rinsed in deionized water, counterstained by Mayer's hematoxylin, and mounted by Eukitt (Bio-Optica, Milan, Italy). Sample reactivity was evaluated by light microscopy (× 200) by two independent observers. Marker positivity was evaluated semi-quantitatively. Staining was evaluated in terms of the localization (nuclear, cytoplasmatic and membrane) of the selected proteins and the percentage of positive cells.

### Clonogenic assay

Following a 72-h exposure to trastuzumab, 500 cells were seeded in 10-cm^2^ dishes in 500 ml of medium. After 14 days, the resulting colonies were fixed and stained using 0.5% crystal violet in 25% methanol; colonies with more than 50 cells were quantified under inverted microscope (Olympus IX51 microscope, Olympus Corporation, Tokyo, Japan) by two independent observers. Five series of samples were prepared for each treatment dose [57].

### Cytofluorimetric analysis

Flow cytometric analysis was performed using a FACS Canto flow cytometer (Becton Dickinson, San Diego, CA, USA) equipped with 488 nm (blue) and 633 (red) lasers. Data acquisition and analysis were performed using FACSDiva (Becton Dickinson) and ModFit 2.0 (DNA Modelling System, Verity Software House, Inc., Topsham, ME, USA). Samples were run in triplicate and 10,000 events were collected for each replica. Data were the average of three experiments, with errors under 5%.

### Cell cycle distribution

After exposure to trastuzumab, cells were fixed in 70% ethanol, stained with propidium iodide (10 mg/ml, MP Biomedicals, Verona, Italy), RNAse (10 kunits/ml, Sigma-Aldrich) and NP40 (0.01%, Sigma Aldrich) overnight at 37C° in the dark, and analyzed by flow cytometry. Data were expressed as fractions of cells in the different cycle phases.

### Bromodeoxyuridine (BrdU) assay

After treatment with trastuzumab100 μg/ml, the cell culture medium was supplemented with 60 μM of BrdU and incubated for an additional 5 h. At the end of the incubation time, cells were fixed, incubated for 25 min with 2M of HCL and then washed with borax 0.1 M. Samples were incubated with anti-BrdU antibody 1:1000 (Sigma-Aldrich) for 60 min. After incubation with FITC-conjugated antibody (goat anti-mouse 1:250, Dako Corporation), cells were stained with 5 mg/ml of propidium iodide for 2 h at 4°C before flow cytometry acquisition.

### Immunophenotypic analysis

Cells were fixed and immunophenotyping was performed using anti-HER2 (1:100) (Invitrogen, Life Technologies, Monza, Italy) antibody for 30 min at 4°C. After three washes, cells were incubated with RPE-conjugated goat anti-rabbit antibody 1:250 (Invitrogen) for 60 min in the dark. Appropriate isotype control was included for each sample.

### Western blot

Cells were treated according to the previously described western blot procedure [58]. The following primary antibodies were used: anti-IQGAP1 (1:400), anti-HER2 (1:800) (Invitrogen, Thermo Fisher Scientific); anti-actin (1:1000) (Sigma-Aldrich); anti-HER4 (1:1000) (Abcam, Cambridge, UK); anti-vinculin (1:1000) (Thermo Fisher Scientific); anti-p27 (1:1000) (BD Biosciences, Milan, Italy); anti-HER3 (1:1000), anti-PTEN (1:1000), anti-mTOR (1:600), anti-MAPK (1:1000), anti-AKT (1:1000), anti-phospho-AKT (Ser^473^) (1:1000) and anti-phospho-HER2 (Tyr^1221/1222^) (1:1000) (Cell Signaling Technology). Precision Plus Protein™ WesternC™ Standards were used as molecular weight standards (Bio-Rad #161-0376). Quantity One Software (Bio-Rad) was used for analysis.

### Small interfering RNA transfection

Silencer^®^ Select Validated siRNA (Ambion, Carlsbad, CA, USA) was utilized for IQGAP1 silencing. A validated Universal Negative ControlTM (Invitrogen) was used as a control for transfection. The siRNA oligonucleotide showing the highest knockdown efficiency of IQGAP1 mRNA in the NCIN87cell line was used for the experiments. Cells were treated according to the previously described procedure [59]. Cells were treated after 72 h.

### RNA extraction and real-time RT-PCR

Total RNA was extracted from cell lines using TRIzol^®^ reagent according to the manufacturer's instructions (Invitrogen). Reverse transcription (RT) reactions were performed using an iScript TM cDNA Synthesis kit (Bio-Rad Laboratories). mRNA expression was analyzed by quantitative Real-Time PCR using the 7500 Real Time PCR system (Applied Biosystems, Thermo Fisher Scientific). The following TaqMan assays (Applied Biosystems, Thermo Fisher Scientific) were used: IQGAP1 (Hs00896595_m1) and its relative gene expression was normalized to glyceraldehyde 3-phosphate dehydrogenase (GAPDH Hs03929097_g1) and β2-microglobulin (Hs00984230_m1). Data showed the average of triplicates ± standard deviation (SD) and were representative of three independent experiments.

### DNA extraction and next-generation sequencing (NGS)

Genomic DNA was extracted using QIAamp DNA MiniKit (Qiagen, Hilden, Germany) as per the manufacturer's protocol. DNA quality was evaluated with *High Sensitivity DNA Analysis Kit* on Bioanalyzer 2100 (Agilent Technologies, Santa Clara, CA, USA) and quantified using Qubit dsDNA BR Assay Kit (Invitrogen). DNA from these cell lines was subjected to target sequencing using a custom panel purchased from Agilent Technologies.

Sequences were achieved by designing primers to capture the entire coding region, exon-intron boundaries (±10 bp) and the promoter region of 4 genes using the Agilent HaloPlex Target Enrichment System (Table 3). Quantified libraries were sequenced on the Illumina MiSeq platform (San Diego, CA, USA) using 2 × 151 bp in pair-end mode and run on an Illumina V2 sequencing flow cell.

**Table 3:** Genes selected for next generation sequencing (NGS) analysis

Gene	entrez ID	cytoband	genomic coordinates	number of exons
*HER2*	2064	17q12	chr17:37844316-37884317	All(31)+5′UTR
*HER3*	2065	12q13.2	chr12:56473788-56495859	All(28)+5′UTR
*HER4*	2066	2q34	chr2:212248319-213403372	All(28)+5′UTR
*IQGAP1*	8826	15q26.1	chr15:90931452-91043360	All(38)

Raw demultiplexed reads from MiSeq sequencer were aligned against the human reference genome hg19 with BWA MEM [60]. GATK version 3.2.2 was used to recalibrate base qualities and realign aligned reads around indels [61]. Regions with coverage of less than or equal to 200x were discarded for downstream analyses. Somatic variant analysis was used to detect mutations: single nucleotide variants (SNVs) were identified using MuTect version 1.1.7 with standard parameters, and GATK IndelGenotyperV2 (with minFraction = 0.01 and minCnt = 5) was used to detect Indels. Genomic and functional annotation of detected variants was made by Annovar [62, 63]. Coverage statistics were performed by Depth of Coverage utility of GATK. BASH and R custom scripts were used to obtain the list of low coverage (200X) regions per sample.

Sequencing runs produced a total of 36,646,538 reads, of which 94.6% mapped on the hg19 human reference genome, with a median coverage depth of 2779, 73X per sample. Only candidate somatic alterations with a read depth of at least 200 and a mutant allele fraction >1% were considered. Sequence results from parental and resistant subclones were compared to identify putative somatic mutations at the basis of the development of resistance.

### Molecular modeling

The UniProt sequences P04626, P21860 and P46940 were taken as a reference for HER2, HER3 ad IQGAP1, respectively. Jalview V. 2.8.2 was used to obtain sequence alignments. The web service Clustal Omega was used with its default settings. Percentage identity coloring was used to generate figures. Three-dimensional figures were generated using an academic version of Maestro software V. 10.1.013.

### Statistical analysis

All experiments were performed at least three times. Quantifiable data were derived from three independent experiments. Statistical analysis was carried out using GRAPH PAD PRISM 5.0 software by applying the Student t test for 2-group comparisons. Differences were considered significant at p<0.05.
